# Association of Plasma Concentration of Vitamin B_12_ With All-Cause Mortality in the General Population in the Netherlands

**DOI:** 10.1001/jamanetworkopen.2019.19274

**Published:** 2020-01-15

**Authors:** Jose L. Flores-Guerrero, Isidor Minović, Dion Groothof, Eke G. Gruppen, Ineke J. Riphagen, Jenny Kootstra-Ros, Anneke Muller Kobold, Eelko Hak, Gerjan Navis, Ron T. Gansevoort, Martin H. de Borst, Robin P. F. Dullaart, Stephan J. L. Bakker

**Affiliations:** 1 University Medical Center Groningen, Division of Nephrology, Department of Internal Medicine, University of Groningen, Groningen, the Netherlands; 2 University Medical Center Groningen, Department of Laboratory Medicine, University of Groningen, Groningen, the Netherlands; 3 Faculty of Science and Engineering, Groningen Research Institute of Pharmacy, University of Groningen, Groningen, the Netherlands; 4 University Medical Center Groningen, Division of Endocrinology, Department of Internal Medicine, University of Groningen, Groningen, the Netherlands

## Abstract

**Importance:**

Higher plasma concentrations of vitamin B_12_ have been associated with mortality in elderly and hospitalized populations, including patients with chronic kidney disease, but the association of plasma concentrations of vitamin B_12_ with mortality in the general population remains unclear.

**Objective:**

To investigate the association of plasma concentrations of vitamin B_12_ with all-cause mortality.

**Design, Setting, and Participants:**

This longitudinal cohort study used post hoc analysis to examine data from participants of the Prevention of Renal and Vascular End-stage Disease Study in Groningen, the Netherlands. Participants included individuals who completed the second screening visit beginning January 1, 2001, excluding those who were missing values of vitamin B_12_ plasma concentrations or used vitamin B_12_ supplementation. Follow-up time was defined between the beginning of the second screening round to end of follow-up on January 1, 2011. Data analysis was conducted from October 2, 2018, to February 22, 2019.

**Exposures:**

Plasma vitamin B_12_ concentration level.

**Main Outcomes and Measures:**

Death as recorded by the Central Bureau of Statistics of Groningen, the Netherlands.

**Results:**

A total of 5571 participants (mean [SD] age, 53.5 [12.0] years; 2830 [50.8%] men) were included in analyses. Median (interquartile range) plasma concentration of vitamin B_12_ was 394.42 (310.38-497.42) pg/mL. During the median (interquartile range) of 8.2 (7.7-8.9) years of follow-up, 226 participants (4.1%) died. According to quartiles of the distribution of plasma vitamin B_12_ concentration levels, mortality rates were 33.8 deaths per 10 000 person-years for the quartile with the lowest plasma concentration of vitamin B_12_ and 65.7 deaths per 10 000 person-years for the quartile with the highest plasma concentration of vitamin B_12_. After adjustment for multiple clinical and laboratory variables, Cox regression analyses found a significant association between higher vitamin B_12_ plasma concentration level and increased risk of all-cause mortality (hazard ratio per 1-SD increase, 1.25 [95% CI, 1.06-1.47]; *P* = .006).

**Conclusions and Relevance:**

These findings suggest that higher levels of plasma concentrations of vitamin B_12_ were associated with increased risk of all-cause mortality after adjusting for age, sex, renal function, and other clinical and laboratory variables. The mechanisms underlying this association remain to be established.

## Key Points

**Question:**

Are plasma concentrations of vitamin B_12_ associated with risk of all-cause mortality among adults from the general population of the Netherlands?

**Findings:**

In this population-based cohort study including 5571 adults, higher plasma concentrations of vitamin B_12_ were associated with a 25% increased adjusted risk of all-cause mortality per 1-SD increase.

**Meaning:**

These findings suggest that higher plasma concentrations of vitamin B_12_ are associated with all-cause mortality, independent of traditional risk factors.

## Teaser

This population-based cohort study examines the association of plasma concentrations of vitamin B_12_ with all-cause mortality among adults in the Netherlands.

## Introduction

Vitamin B_12_ is a hydrosoluble vitamin that plays a substantial role in 1-carbon metabolism. The 1-carbon pathway is involved in several biological functions beyond fetal development, such as mitochondrial metabolism, immune response, and nucleotide homeostasis in nonproliferative tissues.^1^

While the deleterious effects of vitamin B_12_ deficiency, such as anemia, neuropsychiatric symptoms, and other clinical manifestations, are well established,^2^ the potential association of high plasma concentrations of vitamin B_12_ with adverse health outcomes has not been fully explored.^3^ Indeed, a potential association of high vitamin B_12_ plasma concentrations with excess mortality has been assessed in elderly^3,4,5,6,7,8,9^ and hospitalized^10,11^ populations, but it has not been explored in the general population, to our knowledge.

An association of high plasma concentrations of vitamin B_12_ with increased risk of all-cause mortality has been reported among patients undergoing dialysis treatment.^12^ It has also been found that impaired renal function is associated with high plasma concentrations of vitamin B_12_.^13,14^ Furthermore, it has been found that combined supplementation of folic acid, vitamin B_6_, and vitamin B_12_ results in more rapid decline of renal function and an increase in occurrence of vascular events in patients with diabetic nephropathy.^15^ Taken together, these findings underscore the importance of further exploration of a possible role of chronic kidney disease (CKD) in the association of plasma concentrations of vitamin B_12_ with all-cause mortality.

Therefore, this study aimed to assess the association of plasma concentrations of vitamin B_12_ with all-cause mortality in a population-based cohort study. In addition, we aimed to investigate whether findings were further associated with CKD or age, considering that approximately 35% of the elderly population has some degree of CKD.^16^ The Prevention of Renal and Vascular End-stage Disease (PREVEND) study is particularly suitable for such aims, since it has a wide age range and its design is enriched with a CKD component.

## Methods

### Study Cohort

The PREVEND Study is a prospective population-based cohort study in the city of Groningen, the Netherlands. The design of the PREVEND Study has been described in detail elsewhere.^17^ Briefly, from 1997 to 1998, all residents from Groningen aged 28 to 75 years were invited to participate. A total of 40 856 individuals (47.8%) responded to the invitation to participate. From this group, 30 890 individuals had a urinary albumin concentration of less than 10 mg/L and 9966 individuals had a urinary albumin concentration of 10 mg/L or higher in their morning urine sample. After exclusion of individuals with type 1 diabetes and women who were pregnant, 7768 individuals with a urinary albumin concentration of 10 mg/L or higher and a randomly selected control group of 3395 individuals with a urinary albumin concentration of less than 10 mg/L were invited for further investigations in an outpatient clinic. A total of 8592 individuals completed an extensive examination. The PREVEND Study cohort was designed to include any people who met the inclusion criteria, regardless of race/ethnicity, which was recorded according to self-report and included in the analysis owing to potential racial/ethnic disparities in all-cause mortality.^18^

**Figure 1.**
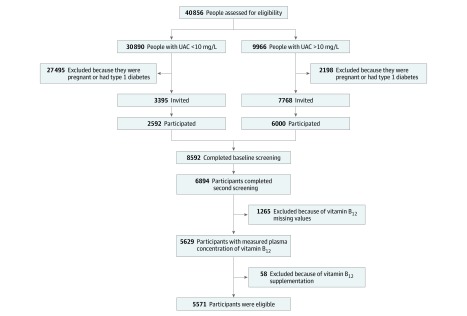
Cohort Recruitment Flowchart UAC indicates urinary albumin concentration.

We used data from 6894 participants who completed the second screening, starting January 1, 2001, excluding 1265 individuals with missing samples for assessment of vitamin B_12_ plasma concentrations and 58 individuals for use of injectable vitamin B_12_ supplementation, leaving a cohort of 5571 participants with complete information for the analysis (Figure 1). In this group, less than 1% of data on laboratory variables was missing. Participants lost to follow-up were considered as censored data. Educational level was categorized into low (ie, no, primary, basic vocational, and secondary education), medium (ie, senior secondary vocational and general senior secondary education), and high (higher professional and higher academic education) according to the International Standard Classification of Education.^19^ Drug dispensing data of injectable vitamin B_12_ supplementation was retrieved from the University Groningen Pharmacy dispensing database IADB.nl.^20^ This report follows the Strengthening the Reporting of Observational Studies in Epidemiology (STROBE) reporting guideline. The protocol for the PREVEND study was approved by the local ethics committee of the University Medical Center Groningen. All participants provided written informed consent, and all procedures were conducted according to the Declaration of Helsinki.^21^

### Laboratory Measurements

Laboratory measurements were performed at the Central Laboratory of the University Medical Center Groningen, the Netherlands. Hematologic measurements, including hemoglobin, hematocrit, and mean corpuscular volume, were measured on a Coulter Counter STKS sample testing system (Coulter Corp) in fresh venous blood according to standard procedures. Plasma glucose level was measured directly after blood sampling. Ethylene diamine tetraacetic acid–anticoagulated plasma samples and sera were stored at −80 °C until analysis.

Vitamin B_12_ plasma concentrations were measured on a Roche platform, using the validated Elecsys Vitamin B_12_ II assay (Roche Diagnostics). The performance of Elecsys Vitamin B_12_ II has been reported in greater detail elsewhere.^22^

Total cholesterol (TC), triglyceride, serum creatinine, and serum cystatin C levels were measured using standard protocols, as described previously.^23,24,25^ Serum ferritin levels were measured using immunoassay, and serum transferrin levels were measured using immunoturbidimetric assay (Roche Diagnostics). Homocysteine concentrations were measured on a Roche Cobas analyzer (Roche Diagnostics). Urinary albumin excretion (UAE) rates were measured as described in two 24-hour urine collections and the results were calculated as a mean for analysis.^25^ The estimated glomerular filtration rates (eGFRs) were calculated using the Chronic Kidney Disease Epidemiology Collaboration combined creatinine–cystatin C equation.^26^

### Clinical Measurements

Height and weight were measured with the participants standing without shoes or heavy outer garments. Body mass index (BMI) was calculated by dividing weight in kilograms by height in meters squared. Systolic and diastolic blood pressure values were recorded as the means of the last 2 recordings of the second visit.

### Ascertainment of End Point

Participants were followed up from the date of the baseline visit until January 1, 2011. Data on mortality were obtained from the municipal register, and the cause of death was obtained by linking the number of the death certificate to the primary cause of death as coded by a physician from the Central Bureau of Statistics.

### Statistical Analysis

Data are presented as the mean (SD) or median (interquartile range [IQR]) for continuous variables and percentages for categorical variables. Cross-sectional group differences among vitamin B_12_ plasma concentration groups at baseline were assessed by Welch 1-way test for normally distributed data, Kruskal-Wallis test for skewed distributed data, and χ^2^ test for categorical variables. Multivariable linear regression analyses were carried out to disclose the associations of vitamin B_12_ plasma concentrations with clinical covariates and laboratory parameters.

For the prospective analysis, we plotted cumulative Kaplan-Meier curves for mortality during follow-up according to quartiles of vitamin B_12_ plasma concentrations. Quartile 1 was defined as vitamin B_12_ plasma concentration less than 338.85 pg/mL (to convert to picomoles per liter, multiply by 0.7378); quartile 2, 338.85 to 397.13 pg/mL; quartile 3, 397.14 to 455.41 pg/mL; and quartile 4, more than 455.41 pg/mL. Time-to-event Cox proportional hazards models were used to assess the hazard ratio (HR) and 95% CI of mortality among 5571 participants with full information at baseline. We calculated HRs in models adjusted for covariates selected on the basis of physiological plausibility and previous literature and organized in cumulative models of clinical significance. The cumulative models depict the influence of different physiological components on the association of vitamin B_12_ plasma concentrations with mortality. The choice for certain confounder adjustments (eg, adjust for systolic but not diastolic blood pressure) was made based on those variables that presented a stronger association on the cross-sectional logistic regression analysis (eTable 1 in the Supplement). The first model was adjusted for age and sex. The second model was further adjusted for relevant clinical variables: race/ethnicity, type 2 diabetes, smoking behavior, alcohol consumption, and education level, which were evaluated as dichotomous variables; BMI, systolic blood pressure, and homocysteine level were evaluated as continuous variables. Model 3 included variables in model 2 plus relevant variables involved in hematological homeostasis, such as ferritin and hemoglobin levels and mean corpuscular volume (continuous variables), which are also associated with plasma concentrations of vitamin B_12_. Model 4 was adjusted for the same variables as model 3 plus TC to high-density lipoprotein cholesterol ratio and glucose level (continuous variables), as a proxy for cardiometabolic risk. Model 5 was adjusted for the variables in model 4 plus history of cancer (dichotomous variable) and history of cardiovascular disease (CVD) (dichotomous variable), given the previous reports of the association of high plasma concentrations of vitamin B_12_ with cancer^27^ and the relevance of CVD in mortality risk. Model 6 was adjusted for the variables included in model 5 plus renal function (ie, eGFR and UAE rate as continuous variables), as this cohort was particularly enriched with participants with a component of CKD. Finally, model 7 was adjusted for the variables in model 6 plus aspartate aminotransferase, aspartate aminotransferase, alkaline phosphatase and γ-glutamyltransferase levels (continuous variables), as previous literature also suggests an important role of hepatic function on the metabolism of vitamin B_12_.^1^ Hazard ratios were computed per 1-SD increment of plasma concentration of vitamin B_12_. Given the right-skewed distribution, vitamin B_12_ plasma concentration data were log_e_ transformed. Hazard ratios were also computed according to vitamin B_12_ plasma concentrations as a categorical variable, with the reference group as quartile 1. To improve the graphic presentation of HRs, quartile 2 and quartile 3 were combined into a single category. Proportionality of hazards assumptions were tested using weighted Schoenfeld residuals for each variable and for every model as a whole. Additionally, interactions with age, eGFR, and UAE rate were analyzed.

Furthermore, we conducted sensitivity analysis consisting of (1) individuals without history of CVD; (2) individuals without history of cancer; (3) individuals without history of vitamin B_12_ deficiency, defined as plasma concentrations of vitamin B_12_ less than 200.60 pg/mL^28^; (4) individuals without history of vitamin B_12_ deficiency judged by plasma concentrations of homocysteine greater than 2.03 mg/L (to convert to micromoles per liter, multiply by 7.397)^28^; (5) all individuals with available information, including those with history of vitamin B_12_ supplementation; and (6) individuals without mild to moderate loss of kidney function (eGFR <60 mL/min/1.73 m^2^). By design, participants with an urinary albumin concentration of 10 mg/L or higher are overrepresented in the PREVEND Study cohort. Therefore, a design-based analysis was performed to take this overselection of participants with elevated UAE rates into account. This statistical weighting method allows conclusions to be generalized to the general population. In addition, all-cause mortality relative risks were estimated for each stratum of alcohol consumption, smoking behavior, and age. Finally, prospective associations of plasma concentrations of vitamin B_12_ with risk of cancer mortality and CVD mortality were assessed.

All statistical tests were 2-sided, and a *P* value less than .05 was considered statistically significant. All statistical analyses were performed with R statistical software version 3.5.1 (R Project for Statistical Computing). Data analysis was conducted from October 2, 2018, to February 22, 2019.

## Results

### Baseline Characteristics

Of 6894 PREVEND Study participants who completed the second round of screening, 5571 participants (mean [SD] age, 53.5 [12.0] years; 2830 [50.8%] men) were included in this study. Participant characteristics at baseline are shown in Table 1. The median (IQR) vitamin B_12_ plasma concentration was 394.42 (310.38-497.42) pg/mL (Table 1). A total of 195 participants (3.5%) had a low vitamin B_12_ plasma concentration (<220.60 pg/mL). After dividing participants by plasma concentration of vitamin B_12_, there were 1390 participants (mean [SD] age, 52.5 [12.3] years; 709 [51.0%] men) in quartile 1, the lowest concentration quartile; 2787 participants (mean [SD] age, 53.4 [11.9] years; 1444 [51.8%] men) in quartiles 2 and 3; and 1394 participants (mean [SD] age, 54.6 [11.6] years; 677 [48.5%] men) in quartile 4, the highest concentration quartile. Participants within the highest quartile of vitamin B_12_ plasma concentrations (>455.41 pg/mL) were more likely to be older and have higher BMI and blood pressure. Additionally, those participants also had higher concentrations of TC and glucose and higher UAE rates. Family history of CKD, history of cancer or CVD, and educational levels were similar across the quartiles of vitamin B_12_ plasma concentrations (Table 1).

**Table 1.** Participant Characteristics According to Quartile of Plasma Concentration of Vitamin B_12_

Characteristic	Participants, No. (%)				*P* Value^a^
	All (N = 5571)	Vitamin B_12_ Plasma Concentration Quartile			
		1 (<338.85 pg/mL) (n = 1390)	2 and 3 (338.85-455.41 pg/mL) (n = 2787)	4 (>455.41 pg/mL) (n = 1394)	
Men	2830 (50.8)	709 (51.0)	1444 (51.8)	677 (48.5)	.12
Age, mean (SD), y	53.5 (12.0)	52.5 (12.3)	53.4 (11.9)	54.6 (11.6)	<.001
White race/ethnicity	5292 (95.0)	1326 (95.4)	2651 (95.1)	1315 (94.3)	.05
Education level^b^					.25
Low	2455 (44.0)	589 (42.3)	1234 (44.3)	632 (45.4)	
Medium	1415 (25.5)	383 (27.7)	697 (25.0)	335 (24.0)	
High	1701 (30.5)	418 (30.0)	856 (3.7)	427 (30.6)	
BMI, mean (SD)	26.7 (4.3)	26.4 (4.1)	26.8 (4.4)	26.5 (4.4)	<.001
Systolic BP, mean (SD), mm Hg	126.3 (18.6)	125.4 (18.4)	126.1 (18.4)	127.8 (19.2)	.001
Diastolic BP, mean (SD), mm Hg	73.5 (9.1)	72.9 (9.1)	73.4 (9.1)	74.2 (9.1)	<.001
Parental history of CKD	25 (0.5)	7 (0.5)	12 (0.4)	7 (0.5)	.74
Parental history of type 2 diabetes	854 (15.3)	203 (14.6)	429 (15.4)	222 (15.9)	.49
Type 2 diabetes	347 (6.2)	55 (3.95)	185 (6.63)	107 (7.67)	<.001
Cancer history	262 (4.7)	71 (5.1)	131 (4.7)	60 (4.3)	.60
CVD history	369 (6.6)	86 (6.2)	180 (6.4)	103 (7.3)	.39
Smoking status					
Never	1577 (28.3)	379 (27.3)	778 (27.9)	420 (30.1)	.01
Former	2378 (42.7)	581 (41.8)	1180 (42.3)	617 (44.3)	
Current	1547 (27.8)	409 (29.4)	796 (28.6)	342 (24.5)	
Alcohol consumption, drinks/wk					
<1	1424 (25.6)	352 (25.3)	707 (25.4)	365 (26.2)	.04
1-7	2653 (47.6)	670 (48.2)	1365 (49.0)	618 (44.3)	
>7	1442 (25.9)	349 (25.1)	694 (24.9)	399 (28.6)	
Using antihypertensive drugs	1404 (19.8)	227 (16.3)	563 (2.2)	314 (22.5)	<.001
Using lipid-lowering drugs	460 (8.2)	93 (6.7)	237 (8.5)	130 (9.3)	.05
Plasma concentration of vitamin B_12_, median (IQR), pg/mL	394.42 (310.38-497.42)	261.59 (226.35-287.34)	394.42 (352.40-439.14)	626.19 (532.66-670.91)	<.001
Ferritin, median (IQR), ng/mL	98.0 (48.0-134.6)	84.0 (44.0-152.2)	101.0 (49.0-174.0)	107.0 (52.0-196.2)	<.001
Transferrin, mean (SD), mg/dL	259 (40)	261 (45)	257 (39)	258 (39)	.03
Hemoglobin, mean (SD), g/dL	13.74 (1.22)	13.65 (1.21)	13.76 (1.22)	13.79 (1.22)	.003
Hematocrit, mean (SD), %	40.88 (3.61)	40.65 (3.53)	4.92 (3.68)	41.03 (3.52)	.01
MCV, mean (SD), μm^3^	90.4 (4.64)	90.8 (4.60)	9.3 (4.64)	90.1 (4.68)	<.001
Homocysteine, mean (SD), mg/L	1.70 (0.59)	1.94 (0.71)	1.67 (0.54)	1.52 (0.48)	<.001
TC, mean (SD), mg/dL	209.27 (40.54)	205.41 (38.61)	209.27 (40.93)	213.90 (40.93)	<.001
HDL-C, mean (SD), mg/dL	48.26 (11.97)	47.10 (11.97)	48.26 (11.97)	49.42 (12.74)	<.001
Triglycerides, median (IQR), mg/dL	99.12 (71.68-142.48)	97.35 (71.68-140.71)	100.00 (71.68-143.36)	98.23 (70.80-146.90)	.69
TC/HDL-C ratio, median (IQR)	4.38 (3.55-5.38)	4.36 (3.60-5.39)	4.40 (3.55-5.37)	4.38 (3.51-5.37)	.70
Glucose, mg/dL	46.49 (79.28-95.50)	84.68 (79.28-93.69)	86.49 (81.08-95.50)	86.49 (81.08-97.30)	<.001
C-reactive protein, median (IQR), mg/L	1.34 (0.61-3.04)	1.18 (0.57-2.88)	1.36 (0.63-3.03)	1.47 (0.62-3.15)	.01
eGFR, mean (SD), mL/min/1.73 m^2^	92.17 (17.10)	92.12 (16.8)	92.5 (17.1)	91.5 (17.2)	<.001
UAE, median (IQR), mg/24 h	8.83 (6.09-16.25)	8.45 (6.00-14.69)	8.73 (6.08-15.90)	9.38 (6.26-18.09)	<.001
ALT, median (IQR), U/L	17.0 (13.0-25.0)	16.0 (12.0-22.0)	18.0 (13.0-25.0)	19.0 (14.0-28.0)	<.001
AST, median (IQR), U/L	22.0 (19.0-26.0)	22.0 (19.0-25.0)	22.0 (19.0-26.0)	23.0 (20.0-28.0)	<.001
ALP, mean (SD), U/L	68.9 (20.3)	67.28 (18.9)	69.20 (2.5)	70.26 (20.9)	<.001
GGT, median (IQR), U/L	24.0 (16.0-39.0)	22.0 (15.0-35.0)	24.0 (16.0-38.0)	27.0 (17.0-46.0)	<.001

Abbreviations: ALP, alkaline phosphatase; ALT, alanine aminotransferase; AST, aspartate aminotransferase; BMI, body mass index (calculated as weight in kilograms divided by height in meters squared); BP, blood pressure; CKD, chronic kidney disease; CVD, cardiovascular disease; eGFR, estimated glomerular filtration rate; GGT, γ-glutamyltransferase; HDL-C, high-density lipoprotein cholesterol; IQR, interquartile range; MCV, mean corpuscular volume; TC, total cholesterol; UAE, urinary albumin excretion.

SI conversion factors: To convert plasma concentration of vitamin B_12_ to picomoles per liter, multiply by 0.7378; ferritin to picomoles per liter, multiply by 2.247; transferrin to micromoles per liter, multiply by 0.123; hemoglobin to grams per liter, multiply by 10; hematocrit to proportion of 1.0, multiply by 0.01; MCV to femtoliters, multiply by 1; homocysteine to micromoles per liter, multiply by 7.397; cholesterol to millimoles per liter, multiply by 0.0259; triglycerides to millimoles per liter, multiply by 0.0113; glucose to millimoles per liter, multiply by 0.0555; C-reactive protein to nanomoles per liter, multiply by 9.524; and ALT, AST, ALP, and GGT to microkatals per liter, multiply by 0.0167.

^a^ *P* values represent the significance of difference across the quartiles of plasmatic vitamin B_12_. *P* values were determined using a 1-way analysis of variance for normally distributed data, Kruskal-Wallis test for skewed distributed data, and χ^2^ test for categorical data.

^b^ Education levels were defined as low, no, primary, basic vocational, and secondary education; medium, senior secondary vocational and general senior secondary education; or high, higher professional and higher academic education.

### Associations at Baseline

The associations of vitamin B_12_ plasma concentrations and other variables of interest were evaluated with univariable and multivariable regression analysis (eTable 1 in the Supplement). In a fully adjusted multivariable model, higher vitamin B_12_ plasma concentration remained positively associated with use of lipid-lowering medication (β = 0.04 [95% CI, 0.01-0.07]; *P* = .04), high-density lipoprotein cholesterol (β = 0.09 [95% CI, 0.05-0.13]; *P* < .001), ferritin (β = 0.05 [95% CI, 0.02-0.09]; *P* = .005), and hemoglobin (β = 0.15 [95% CI, 0.04-0.27]; *P* = .009) levels and inversely associated with mean corpuscular volume (β = −0.09 [95% CI, −0.12 to −0.05]; *P* < .001), homocysteine level (β = −0.34 [95% CI, −0.38 0.30]; *P* < .001), and eGFR (β = −0.11 [95% CI, −0.16, to −0.07]; *P* < .001).

### Longitudinal Analysis

**Figure 2.**
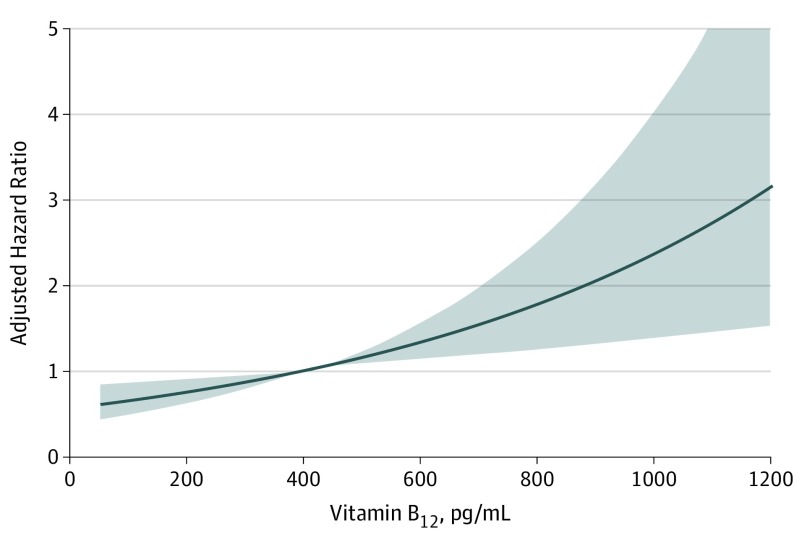
Association of Plasma Concentration of Vitamin B_12_ With Adjusted Risk of All-Cause Mortality To convert plasma concentration of vitamin B_12_ to picomoles per liter, multiply by 0.7378. Shading indicates 95% CI.

**Figure 3.**
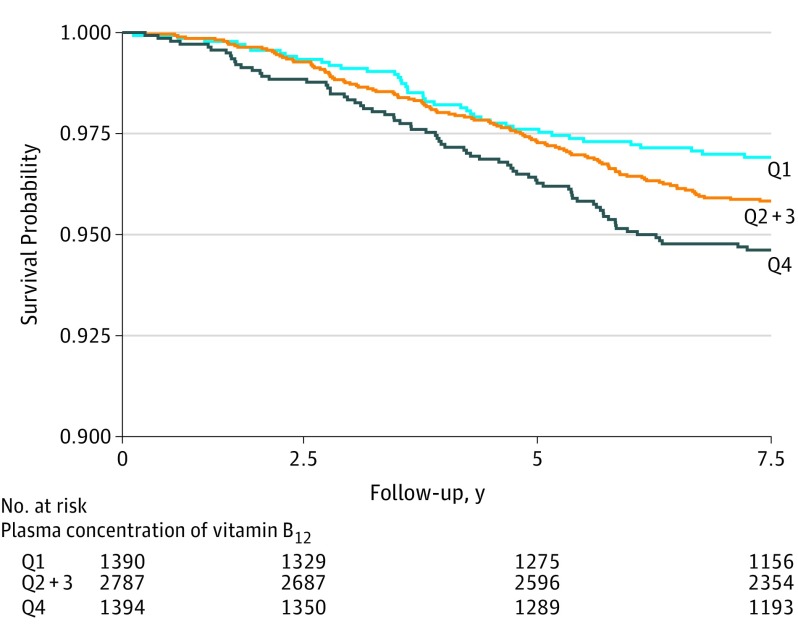
Kaplan-Maier Plot for Vitamin B_12_ Plasma Concentration and All-Cause Mortality Quartile (Q) 1 indicates vitamin B_12_ plasma concentration less than 338.85 pg/mL (to convert to picomoles per liter, multiply by 0.7378); Q2 and 3, vitamin B_12_ plasma concentration 338.85 to 455.41 pg/mL; and Q4, vitamin B_12_ plasma concentration 455.41 pg/mL or higher.

During median (IQR) follow-up of 8.2 (7.7-8.9) years, 226 participants (4.1%) died. Higher plasma concentrations of vitamin B_12_, when analyzed as HR per 1 log_e_ SD increase, were associated with mortality after full adjustment (adjusted HR, 1.25 [95% CI, 1.06-1.47]; *P* = .006) (Table 2 and Figure 2). Kaplan-Meier curves for mortality according to quartiles of vitamin B_12_ plasma concentration are presented in Figure 3. There was an increased risk of all-cause mortality associated with the top quartile of vitamin B_12_ concentrations (*P* for log-rank test <.001). In age- and sex-adjusted Cox regression analysis that examined the vitamin B_12_ plasma concentration as a categorical variable with quartile 1 as the reference group, the fourth quartile of vitamin B_12_ plasma concentrations was associated with increased risk of mortality (HR, 1.73 [95% CI, 1.18-2.53]; *P* = .005) (Table 2). The association remained significant after full adjustment (adjusted HR, 1.85 [95% CI, 1.16-2.97]; *P* = .01) (Table 2). The proportional hazards assumptions were not violated for any of the variables in the full model. The interaction terms of vitamin B_12_ plasma concentration with age, eGFR, and UAE rate with all-cause mortality were not significant when included in either the crude or the sex- and age-adjusted models.

**Table 2.** Associations of Plasma Concentration of Vitamin B_12_ With Risk of All-Cause Mortality

Model	Vitamin B_12_ Plasma Concentration						
	Per 1-SD Increment		Quartile 1 (<338.85 pg/mL)	Quartiles 2 and 3 (338.85-455.41 pg/mL)		Quartile 4 (>455.41 pg/mL)	
	HR (95% CI)	*P* Value		HR (95% CI)	*P* Value	HR (95% CI)	*P* Value
Participants, No.	5571		1390	2787		1394	
Deaths, No.	226		41	112		73	
Unadjusted	1.22 (1.07-1.40)	.002	1 [Reference]	1.35 (0.94-1.93)	.10	1.76 (1.20-2.58)	.003
Model 1^a^	1.22 (1.07-1.39)	.003	1 [Reference]	1.34 (0.94-1.92)	.10	1.73 (1.18-2.53)	.005
Model 2^b^	1.25 (1.09-1.44)	.001	1 [Reference]	1.34 (0.93-1.94)	.12	1.84 (1.23-2.76)	.002
Model 3^c^	1.26 (1.09-1.47)	.002	1 [Reference]	1.38 (0.93-2.04)	.10	1.77 (1.15-2.72)	.009
Model 4^d^	1.26 (1.08-1.46)	.003	1 [Reference]	1.38 (0.93-2.06)	.10	1.72 (1.11-2.67)	.01
Model 5^e^	1.24 (1.07-1.44)	.005	1 [Reference]	1.38 (0.93-2.06)	.10	1.70 (1.09-2.63)	.01
Model 6^f^	1.25 (1.06-1.47)	.006	1 [Reference]	1.41 (0.93-2.15)	.10	1.84 (1.15-2.94)	.01
Model 7^g^	1.25 (1.06-1.47)	.006	1 [Reference]	1.38 (0.91-2.10)	.12	1.85 (1.16-2.97)	.01

Abbreviation: HR, hazard ratio.

SI conversion factor: To convert plasma concentration of vitamin B_12_ to picomoles per liter, multiply by 0.7378.

^a^ Adjusted for age and sex.

^b^ Adjusted for model 1, ethnicity, body mass index, type 2 diabetes, smoking status (ie, never, past, or current), alcohol consumption (ie, <1, 1-7, or >7 drinks/week), education (ie, low, medium, or high), systolic blood pressure, and homocysteine level.

^c^ Adjusted for model 2, ferritin level, hemoglobin, and mean corpuscular volume.

^d^ Adjusted for model 3, total cholesterol to high-density lipoprotein cholesterol ratio, and glucose level.

^e^ Adjusted for model 4, history of cancer, and history of cardiovascular disease.

^f^ Adjusted for model 5, estimated glomerular filtration rate, and urinary albumin excretion rate.

^g^ Adjusted for model 6, alanine aminotransferase, aspartate aminotransferase, alkaline phosphatase, and γ-glutamyltransferase levels.

### Sensitivity Analyses

Excluding patients with a history of CVD, cancer, low plasma concentrations of vitamin B_12_, or high plasma concentrations of homocysteine did not materially change the results (eTables 2, 3, 4, and 5 in the Supplement). Inclusion of participants with history of vitamin B_12_ supplementation also did not materially change the results (eTable 6 in the Supplement). In the analysis conducted after exclusion of individuals with mild to moderate loss of kidney function (eGFR <60 mL/min/1.73 m^2^), there was no association of plasma concentration of vitamin B_12_ with all-cause mortality after adjustment for history of CVD (eTable 7 in the Supplement). Nonetheless, the results of the design-based analysis are in line with the main results presented in Table 2 (eTable 8 in the Supplement). The relative risk of all-cause mortality across the different strata of smoking behavior (eTable 9 in the Supplement), alcohol consumption (eTable 10 in the Supplement), and age (eTable 11 in the Supplement) showed a consistent association of higher plasma concentration of vitamin B_12_ with increased risk of all-cause mortality. We found no independent associations of plasma concentration of vitamin B_12_ with cancer mortality (eTable 12 in the Supplement) or with CVD mortality (eTable 13 in the Supplement).

## Discussion

In this prospective population-based cohort study, we investigated the associations of plasma concentration of vitamin B_12_ with all-cause mortality. Baseline characteristics, such as older age, high blood pressure, reduced eGFR, and elevated UAE rate, as well as increased concentrations of liver enzymes, were positively associated with higher plasma concentrations of vitamin B_12_, in agreement with findings from other studies.^3,8,11^ While the cross-sectional associations as found in this study do not provide an insight for a particular cause of death, we observed that high circulating vitamin B_12_ plasma concentrations were associated with significantly higher risk of all-cause mortality. The association remained significant after adjustment for established risk factors, including age, sex, BMI, type 2 diabetes, tobacco use, and alcohol consumption, as well as biomarkers associated with renal and liver function. We found no indication of change in this association by either renal function or age.

In our study, the participants in the highest quartile of vitamin B_12_ had a mean age 1 year older than the total cohort’s mean age (53.5 years). However, the association we found of plasma concentration of vitamin B_12_ with all-cause mortality was independent of age. Previous studies have explored such an association only in elderly people. A study by Salles et al^6^ reported an association of high vitamin B_12_ plasma concentration with increased mortality risk among elderly individuals (mean age, 86 years). On the other hand, a study by Robinson et al^5^ reported that vitamin B_12_ plasma concentration levels were not associated with death risk in an elderly population. Moreover, in line with previous reports, we did not find a statistically significant difference between sexes on the association of vitamin B_12_ plasma concentration with all-cause mortality.

Contrary to the findings reported in a study by Arendt et al,^27^ we did not find a significant association of vitamin B_12_ plasma concentration with risk of cancer mortality. Moreover, there was no cross-sectional association between concentrations of vitamin B_12_ plasma concentration and cancer history at baseline. In addition, the prospective association of vitamin B_12_ plasma concentration with all-cause mortality was not changed by adjustment for history of cancer at baseline. Also in sensitivity analyses, the association of plasma concentration of vitamin B_12_ with all-cause mortality persisted after exclusion of participants with history of cancer.

Likewise, the association of all-cause mortality with high plasma concentrations of vitamin B_12_ could not be attributed to CVD. We demonstrated that adjustment for blood pressure, as well as lipid profile and type 2 diabetes, did not change the association. Furthermore, in sensitivity analyses conducted after exclusion of participants with history of CVD, the association remained. In addition, plasma concentration of vitamin B_12_ was not associated with risk of CVD mortality. Such results are in line with a 12-year follow-up study from 2019^29^ that found that plasma concentrations of vitamin B_12_ were not associated with the incidence risk of atherosclerotic disease.

Considering the universal role of the 1-carbon pathway in mammals, the explanation of the described association seems to rely in the role of vitamin B_12_ in the homeostasis of nonproliferative tissues^6^ rather than proliferative tissues,^1^ such as the bone marrow and other hematopoietic tissues, as we demonstrated that this association was independent of cancer history.

To date, the underlying mechanism of the association of plasma concentration of vitamin B_12_ with mortality is incompletely understood, to our knowledge. The proposed mechanisms to explain the association are that high vitamin B_12_ plasma concentrations may represent a response to increased release of vitamin B_12_ from liver storage, decreased clearance, upregulation of haptocorrin and transcobalamin synthesis, or diminished affinity of vitamin B_12_ for transporter proteins.^1,3^ Those situations are often present as a consequence of liver damage or CKD, which could be represented by the baseline association of high plasma concentrations of vitamin B_12_ with elevated concentrations of hepatic enzymes. Nonetheless, a definite mechanism has not been described, to our knowledge.^14,30^

The results of this study could also be clinically interpreted in the context of oral vitamin supplementation. Concern about the excess intake of vitamins, particularly vitamin B_12_, has gained attention. A 2010 study by Løland et al^31^ reported that vitamin B supplementation had no beneficial effect on the progression of coronary artery disease, as had been hypothesized previously. Moreover, in a prospective study with 75 864 women,^32^ vitamin B_12_ supplementation was associated with an increased risk of hip fracture. In that sense, our results may also suggest that caution should be taken when considering vitamin B_12_ supplementation in the absence of vitamin B_12_ deficiency.

### Strengths and Limitations

This study has several strengths. To our knowledge, this is the first study reporting on the association of all-cause mortality with higher vitamin B_12_ plasma concentrations in the general population in which the vitamin B_12_ plasma concentration measurement was performed in unselected individuals, as we excluded participants with B_12_ supplementation, which is one source of bias in other studies.^27^ Moreover, we adjusted our results for several confounding variables, including liver function parameters, and found that the association remained, whereas other authors have reported loss of significance after such adjustment.^11^ Another strength of this study is the implementation of a robust method of plasma concentration of vitamin B_12_ quantification. The accuracy of the assay that we used has been evaluated using the vitamin B_12_ World Health Organization International Standard.^33^ Moreover, it has been reported that the Elecsys Vitamin B_12_ assay is not affected by anti–intrinsic factor antibodies, enhancing the reliability of this assay.^34^ Furthermore, this is the largest study to date reporting on such an association, to our knowledge, which enabled us to carry out sufficiently powered multivariable adjusted analyses and testing the robustness of the findings using several sensitivity analyses to provide solid evidence. Finally, the PREVEND cohort was enriched for increased albumin excretion. We therefore conducted design-based analyses, making our results valid for the general population.

Several limitations of this study also need to be addressed. First, the PREVEND cohort study mainly comprises individuals of European ancestry, which could limit extrapolation of our findings to other races/ethnicities. Second, we did not have measurements of vitamin B_12_ plasma concentrations beyond baseline assessment, which limited us to evaluate the regression dilution of vitamin B_12_. Third, we only had access to pharmacy records on injectable vitamin B_12_ supplementation, but not for over-the-counter tablets, which could limit the implication of cobalamin supplementation; likewise, we had no data on reason for performing the vitamin B_12_ injections. Furthermore, it is worth noting that residual confounding is an important limitation in all observational studies. To further evaluate the associations of well-known mortality risk factors, we provided a supplementary analysis of all-cause mortality relative risks for each stratum of alcohol consumption as well as smoking behavior. Additionally, dietary patterns can also be influence plasma concentration of vitamin B_12_ and risk of mortality. In the PREVEND cohort, detailed dietary information was not available, which we consider a limitation. Therefore, the possibility of a noncausal association should not be discarded and deserves further investigation.

## Conclusions

In this population-based cohort study, high plasma concentrations of vitamin B_12_ were associated with an increased risk of all-cause mortality. The prospective association was independent of age, renal function, and other comorbidities, such as history of cancer. Further investigation is needed to unravel the complexity of 1-carbon metabolism in different mortality causes, such as cardiometabolic disease and cancer.

## Notes

Supplement. **eTable 1.** Multivariable Linear Regression Analyses With Plasma Concentration of Vitamin B_12_ as the Dependent Variable **eTable 2.** Prospective Associations of Plasma Concentration of Vitamin B_12_ With Risk of All-Cause Mortality After Excluding Individuals With History of Cardiovascular Disease **eTable 3.** Prospective Associations of Plasma Concentration of Vitamin B_12_ With Risk of All-Cause Mortality After Excluding Individuals With History of Cancer **eTable 4.** Prospective Associations of Plasma Concentration of Vitamin B_12_ With Risk of All-Cause Mortality After Excluding Individuals With Low Plasma Concentrations Vitamin B_12_ **eTable 5.** Prospective Associations of Plasma Concentration of Vitamin B_12_ With Risk of All-Cause Mortality After Excluding Individuals With High Plasma Concentrations Homocysteine **eTable 6.** Prospective Associations of Plasma Concentration of Vitamin B_12_ With Risk of All-Cause Mortality in All Individuals With Available Information **eTable 7.** Prospective Associations of Plasma Concentration of Vitamin B_12_ With Risk of All-Cause Mortality After Excluding Individuals With Mild to Moderated Loss of Kidney Function **eTable 8.** Prospective Associations of Plasma Concentration of Vitamin B_12_ With Risk of All-Cause Mortality in a Design-Based Analysis **eTable 9.** All-Cause Mortality Relative Risks for Each Stratum of Smoking Behavior **eTable 10.** All-Cause Mortality Relative Risks for Each Stratum of Alcohol Consumption Behavior **eTable 11.** All-Cause Mortality Relative Risks for Each Stratum of Age **eTable 12.** Prospective Associations of Plasma Concentration of Vitamin B_12_ With Risk of Cancer Mortality **eTable 13.** Prospective Associations of Plasma Concentration of Vitamin B_12_ With Risk of Cardiovascular Mortality
