# Supplementary material for: Association of Plasma Concentration of Vitamin B12 With All-Cause Mortality in the General Population in the Netherlands
Source: JAMA Netw Open. 2020 Jan 15;3(1):e1919274. doi: 10.1001/jamanetworkopen.2019.19274 (PMC6991261; doi:10.1001/jamanetworkopen.2019.19274)
Supplement: Supplement. — eTable 1. Multivariable Linear Regression Analyses With Plasma Concentration of Vitamin B12 as the Dependent Variable eTable 2. Prospective Associations of Plasma Concentration of Vitamin B12 With Risk of All-Cause Mortality After Excluding Individuals With History of Cardiovascular Disease eTable 3. Prospective Associations of Plasma Concentration of Vitamin B12 With Risk of All-Cause Mortality After Excluding Individuals With History of Cancer eTable 4. Prospective Associations of Plasma Concentration of Vitamin B12 With Risk of All-Cause Mortality After Excluding Individuals With Low Plasma Concentrations Vitamin B12 eTable 5. Prospective Associations of Plasma Concentration of Vitamin B12 With Risk of All-Cause Mortality After Excluding Individuals With High Plasma Concentrations Homocysteine eTable 6. Prospective Associations of Plasma Concentration of Vitamin B12 With Risk of All-Cause Mortality in All Individuals With Available Information eTable 7. Prospective Associations of Plasma Concentration of Vitamin B12 With Risk of All-Cause Mortality After Excluding Individuals With Mild to Moderated Loss of Kidney Function eTable 8. Prospective Associations of Plasma Concentration of Vitamin B12 With Risk of All-Cause Mortality in a Design-Based Analysis eTable 9. All-Cause Mortality Relative Risks for Each Stratum of Smoking Behavior eTable 10. All-Cause Mortality Relative Risks for Each Stratum of Alcohol Consumption Behavior eTable 11. All-Cause Mortality Relative Risks for Each Stratum of Age eTable 12. Prospective Associations of Plasma Concentration of Vitamin B12 With Risk of Cancer Mortality eTable 13. Prospective Associations of Plasma Concentration of Vitamin B12 With Risk of Cardiovascular Mortality [file jamanetwopen-3-e1919274-s001.pdf]

Supplementary Online Content

Flores-Guerrero JL, Minović I, Groothof D, et al. Association of plasma concentration of vitamin B_{12} with all-cause mortality in the general population in the Netherlands. *JAMA Netw Open*. 2020;3(1):e1919274. doi:10.1001/jamanetworkopen.2019.19274

eTable 1. Multivariable Linear Regression Analyses With Plasma Concentration of Vitamin B_{12} as the Dependent Variable

eTable 2. Prospective Associations of Plasma Concentration of Vitamin B_{12} With Risk of All-Cause Mortality After Excluding Individuals With History of Cardiovascular Disease

eTable 3. Prospective Associations of Plasma Concentration of Vitamin B_{12} With Risk of All-Cause Mortality After Excluding Individuals With History of Cancer

eTable 4. Prospective Associations of Plasma Concentration of Vitamin B_{12} With Risk of All-Cause Mortality After Excluding Individuals With Low Plasma Concentrations Vitamin B_{12}

eTable 5. Prospective Associations of Plasma Concentration of Vitamin B_{12} With Risk of All-Cause Mortality After Excluding Individuals With High Plasma Concentrations Homocysteine

eTable 6. Prospective Associations of Plasma Concentration of Vitamin B_{12} With Risk of All-Cause Mortality in All Individuals With Available Information

eTable 7. Prospective Associations of Plasma Concentration of Vitamin B_{12} With Risk of All-Cause Mortality After Excluding Individuals With Mild to Moderated Loss of Kidney Function

eTable 8. Prospective Associations of Plasma Concentration of Vitamin B_{12} With Risk of All-Cause Mortality in a Design-Based Analysis

eTable 9. All-Cause Mortality Relative Risks for Each Stratum of Smoking Behavior

eTable 10. All-Cause Mortality Relative Risks for Each Stratum of Alcohol Consumption Behavior

eTable 11. All-Cause Mortality Relative Risks for Each Stratum of Age

eTable 12. Prospective Associations of Plasma Concentration of Vitamin B_{12} With Risk of Cancer Mortality

eTable 13. Prospective Associations of Plasma Concentration of Vitamin B_{12} With Risk of Cardiovascular Mortality

This supplementary material has been provided by the authors to give readers additional information about their work.

© 2020 Flores-Guerrero JL et al. *JAMA Network Open.*

eTable 1. Multivariable Linear Regression Analyses With Plasma Concentration of Vitamin B_{12} as the Dependent Variable

**Variables**	**b**	**b (95% CI)**	**β**	**β (95% CI)**	***P*-value**
Sex	0.92	[-9.97, 11.81]	0.00	[-0.04, 0.05]	0.86
Age	0.14	[-0.35, 0.63]	0.01	[-0.04, 0.06]	0.58
Ethnicity	-10.49	[-29.20, 8.22]	-0.02	[-0.05, 0.01]	0.27
Education (high)	0.74	[-7.89, 9.38]	0.00	[-0.03, 0.04]	0.86
BMI, kg/m^2	-0.58	[-1.59, 0.43]	-0.02	[-0.06, 0.02]	0.26
DBP, mm Hg	-0.16	[-0.48, 0.17]	-0.03	[-0.08, 0.03]	0.35
SBP, mm Hg	0.62	[-0.02, 1.25]	0.05	[0.00, 0.10]	0.05
Parental history of CKD	-7.70	[-55.20, 39.80]	-0.01	[-0.04, 0.03]	0.75
Parental history of T2D	-1.92	[-11.97, 8.14]	-0.01	[-0.04, 0.03]	0.70
T2D	5.47	[-16.78, 27.71]	0.01	[-0.03, 0.05]	0.62
Cancer history	2.40	[-15.54, 20.34]	0.00	[-0.03, 0.04]	0.79
CVD history	1.48	[-16.45, 19.40]	0.00	[-0.03, 0.04]	0.87
Smoking	-0.59	[-3.98, 2.80]	-0.01	[-0.04, 0.03]	0.73
Alcohol intake	-3.93	[-9.60, 1.74]	-0.02	[-0.06, 0.01]	0.17
Antihypertensive drugs	8.63	[-2.11, 19.37]	0.03	[-0.01, 0.07]	0.11
Lipid-lowering drugs	15.42	[0.60, 30.25]	0.04	[0.01, 0.07]	0.04*
Ferritin, (µg/L)	0.05	[0.01, 0.08]	0.05	[0.02, 0.09]	0.005**
Transferrin, (g/L)	-12.62	[-22.66, -2.58]	-0.04	[-0.08, -0.01]	0.01*
Hemoglobin, mmol/L	23.95	[5.85, 42.04]	0.15	[0.04, 0.27]	0.009**
Hematocrit, (%)	-333.02	[-695.34, 29.30]	-0.10	[-0.21, 0.01]	0.07
MCV, (fL)	-2.18	[-3.11, -1.25]	-0.09	[-0.12, -0.05]	<0.001***
Homocysteine, (µmol/L)	-9.02	[-10.00, -8.04]	-0.34	[-0.38, -0.30]	<0.001***
TC, mmol/L	2.60	[-1.60, 6.79]	0.02	[-0.01, 0.06]	0.22
HDL-C, mmol/L	32.88	[17.58, 48.18]	0.09	[0.05, 0.13]	<0.001***
TG, mmol/L	-5.60	[-10.73, -0.47]	-0.04	[-0.08, -0.00]	0.03*
Glucose, mmol/L	3.04	[-1.69, 7.76]	0.03	[-0.02, 0.07]	0.20
CRP, mg/L	0.26	[-0.44, 0.95]	0.01	[-0.02, 0.05]	0.47
eGFR, mL/min/1.73m^2	-0.78	[-1.11, -0.46]	-0.11	[-0.16, -0.07]	<0.001***
UAE, mg/24h	0.01	[-0.01, 0.04]	0.02	[-0.01, 0.05]	028
ALT, (U/L)	0.66	[0.23, 1.09]	0.08	[0.03, 0.13]	0.002**
AST, (U/L)	0.74	[0.05, 1.42]	0.05	[0.01, 0.10]	0.03*
ALP, (U/L)	0.18	[-0.03, 0.38]	0.03	[0.00, 0.07]	0.08
GGT, (U/L)	0.20	[0.06, 0.33]	0.06	[0.02, 0.10]	0.005**

Standardized (β) and unstandardized (b) regression coefficients are shown. Significance codes: '***': < 0.001; '**': < 0.01; '*': < 0.05.

© 2020 Flores-Guerrero JL et al. *JAMA Network Open.*

eTable 2. Prospective Associations of Plasma Concentration of Vitamin B_{12} With Risk of All-Cause Mortality After Excluding Individuals With History of Cardiovascular Disease

	**Vitamin B_{12} Per 1 SD Increment**		**Q1**	**Q2+Q3**		**Q4**	
Participants, *n*	5202		1286	2607		1309	
Events, *n*	167		29	83		55	
	**HR (95 % CI)**	***P*-Value**		**HR (95 % CI)**	***P*-Value**	**HR (95 % CI)**	***P*-Value**
Crude Model	1.32 [1.13;1.53]	<0.001	(ref)	1.40 [0.91;2.13]	0.12	1.84 [1.17;2.88]	0.007
Model 1	1.32 [1.13;1.55]	<0.001	(ref)	1.37 [0.90;2.09]	0.14	1.83 [1.16;2.87]	0.008
Model 2	1.35 [1.14;1.59]	< 0.001	(ref)	1.29 [0.83;2.00]	0.25	1.86 [1.15;2.98]	0.01
Model 3	1.42 [1.20;1.69]	< 0.001	(ref)	1.40 [0.88;2.22]	0.15	2.05 [1.24;3.38]	0.005
Model 4	1.40 [1.17;1.67]	< 0.001	(ref)	1.38 [0.86;2.22]	0.18	1.93 [1.16;3.23]	0.01
Model 5	1.40 [1.17;1.67]	< 0.001	(ref)	1.36 [0.85;2.18]	0.20	1.90 [1.13;3.18]	0.01
Model 6	1.42 [1.18;1.71]	< 0.001	(ref)	1.43 [0.87;2.37]	0.16	2.13 [1.23;3.69]	0.006
Model 7	1.41 [1.17;1.71]	< 0.001	(ref)	1.44 [0.87;2.37]	0.15	2.12 [1.22;3.68]	0.007

Data are presented as hazard ratios (HRs) with 95% confidence intervals (CIs). For HR of Vitamin B_{12} per 1 SD increment, data was $\log_e$ transformed.

Model 1: Model adjusted for age and sex

Model 2: Model 1 + ethnicity + BMI + T2D + Smoking status (never, past, current) + Alcohol consumption (<1, 1-7,>7 drinks/week) + Education (low, medium, high) + SBP + homocysteine

Model 3: Model 2 + ferritin + hemoglobin + MCV

Model 4: Model 3 + TC / HDL-C ratio + glucose

Model 5: Model 4 + cancer history

Model 6: Model 5 + eGFR + UAE

Model 7: Model 6 + ALT + AST + ALP + GGT

© 2020 Flores-Guerrero JL et al. *JAMA Network Open.*

eTable 3. Prospective Associations of Plasma Concentration of Vitamin B_{12} With Risk of All-Cause Mortality After Excluding Individuals With History of Cancer

	**Vitamin B_{12} Per 1 SD Increment**		**Q1**	**Q2+Q3**		**Q4**	
Participants, *n*	5308		1319	2655		1334	
Events, *n*	203		36	101		66	
	**HR (95 % CI)**	***P*-Value**		**HR (95 % CI)**	***P*-Value**	**HR (95 % CI)**	***P*-Value**
Crude Model	1.21 [1.06;1.39]	0.006	(ref)	1.38 [0.94;2.02]	0.09	1.79 [1.19;2.69]	0.004
Model 1	1.21 [1.05;1.39]	0.008	(ref)	1.37 [0.93;2.00]	0.10	1.75 [1.17;2.63]	0.007
Model 2	1.25 [1.07;1.44]	0.003	(ref)	1.35 [0.91;1.99]	0.13	1.91 [1.25;2.92]	0.002
Model 3	1.25 [1.07;1.47]	0.005	(ref)	1.42 [0.93;2.15]	0.10	1.84 [1.17;2.92]	0.008
Model 4	1.25 [1.06;1.47]	0.007	(ref)	1.42 [0.93;2.17]	0.10	1.81 [1.13;2.87]	0.01
Model 5	1.23 [1.05;1.45]	0.01	(ref)	1.40 [0.92;2.13]	0.12	1.75 [1.10;2.80]	0.01
Model 6	1.23 [1.04;1.46]	0.01	(ref)	1.46 [0.93;2.27]	0.09	1.90 [1.16;3.11]	0.01
Model 7	1.23 [1.04;1.46]	0.01	(ref)	1.45 [0.93;2.27]	0.09	1.91 [1.16;3.13]	0.01

Data are presented as hazard ratios (HRs) with 95% confidence intervals (CIs). For HR of Vitamin B_{12} per 1 SD increment, data was $\log_e$ transformed.

Model 1: Model adjusted for age and sex

Model 2: Model 1 + ethnicity + BMI + T2D + Smoking status (never, past, current) + Alcohol consumption (<1, 1-7,>7 drinks/week) + Education (low, medium, high) + SBP + homocysteine

Model 3: Model 2 + ferritin + hemoglobin + MCV

Model 4: Model 3 + TC / HDL-C ratio + glucose

Model 5: Model 4 + CVD history

Model 6: Model 5 + eGFR + UAE

Model 7: Model 6 + ALT + AST + ALP + GGT

© 2020 Flores-Guerrero JL et al. *JAMA Network Open.*

eTable 4. Prospective Associations of Plasma Concentration of Vitamin B_{12} With Risk of All-Cause Mortality After Excluding Individuals With Low Plasma Concentrations Vitamin B_{12}

	**Vitamin B_{12} Per 1 SD Increment**		**Q1**	**Q2+Q3**		**Q4**	
Participants, *n*	5376		1344	2685		1347	
Events, *n*	217		36	111		70	
	**HR (95 % CI)**	***P*-Value**		**HR (95 % CI)**	***P*-Value**	**HR (95 % CI)**	***P*-Value**
Crude Model	1.27 [1.12;1.45]	<0.001	(ref)	1.52 [1.04;2.21]	0.02	1.91 [1.28;2.86]	0.001
Model 1	1.26 [1.11;1.44]	<0.001	(ref)	1.47 [1.01;2.14]	0.04	1.87 [1.25;2.80]	0.002
Model 2	1.29 [1.13;1.48]	< 0.001	(ref)	1.48 [1.01;2.19]	0.04	2.00 [1.31;3.05]	0.001
Model 3	1.29 [1.12;1.50]	< 0.001	(ref)	1.47 [0.98;2.21]	0.06	1.87 [1.19;2.93]	0.006
Model 4	1.28 [1.10;1.48]	0.001	(ref)	1.45 [0.96;2.17]	0.07	1.79 [1.14;2.81]	0.01
Model 5	1.26 [1.08;1.46]	0.002	(ref)	1.43 [0.96;2.15]	0.08	1.75 [1.11;2.76]	0.01
Model 6	1.28 [1.09;1.49]	0.002	(ref)	1.46 [0.95;2.26]	0.08	1.93 [1.19;3.12]	0.007
Model 7	1.28 [1.10;1.50]	0.001	(ref)	1.44 [0.93;2.22]	0.09	1.93 [1.19;3.13]	0.007

Data are presented as hazard ratios (HRs) with 95% confidence intervals (CIs). For HR of Vitamin B_{12} per 1 SD increment, data was $\log_e$ transformed.

Model 1: Model adjusted for age and sex

Model 2: Model 1 + ethnicity + BMI + T2D + Smoking status (never, past, current) + Alcohol consumption (<1, 1-7,>7 drinks/week) + Education (low, medium, high) + SBP + homocysteine

Model 3: Model 2 + ferritin + hemoglobin + MCV

Model 4: Model 3 + TC / HDL-C ratio + glucose

Model 5: Model 4 + cancer history + CVD history

Model 6: Model 5 + eGFR + UAE

Model 7: Model 6 + ALT + AST + ALP + GGT

© 2020 Flores-Guerrero JL et al. *JAMA Network Open.*

eTable 5. Prospective Associations of Plasma Concentration of Vitamin B_{12} With Risk of All-Cause Mortality After Excluding Individuals With High Plasma Concentrations Homocysteine

	**Vitamin B_{12} Per 1 SD Increment**		**Q1**	**Q2+Q3**		**Q4**	
Participants, *n*	4222		1055	2105		1062	
Events, *n*	120		17	63		40	
	**HR (95 % CI)**	***P*-Value**		**HR (95 % CI)**	***P*-Value**	**HR (95 % CI)**	***P*-Value**
Crude Model	1.38 [1.16;1.65]	<0.001	(ref)	1.85 [1.08;3.15]	0.02	2.33 [1.32;4.11]	0.003
Model 1	1.26 [1.05;1.51]	0.01	(ref)	1.47 [0.86;2.51]	0.15	1.79 [1.01;3.16]	0.04
Model 2	1.23 [1.03;1.48]	0.02	(ref)	1.43 [0.84;2.46]	0.18	1.73 [0.98;3.06]	0.06
Model 3	1.24 [1.02;1.51]	0.03	(ref)	1.22 [0.71;2.12]	0.47	1.57 [0.87;2.84]	0.13
Model 4	1.23 [1.00;1.50]	0.04	(ref)	1.23 [0.71;2.13]	0.45	1.52 [0.84;2.77]	0.17
Model 5	1.21 [0.99;1.48]	0.04	(ref)	1.24 [0.72;2.15]	0.44	1.46 [0.80;2.66]	0.21
Model 6	1.24 [1.01;1.53]	0.03	(ref)	1.25 [0.71;2.21]	0.43	1.58 [0.85;2.93]	0.14
Model 7	1.25 [1.02;1.54]	0.03	(ref)	1.27 [0.72;2.25]	0.41	1.64 [0.88;3.05]	0.12

Data are presented as hazard ratios (HRs) with 95% confidence intervals (CIs). For HR of Vitamin B_{12} per 1 SD increment, data was $\log_e$ transformed.

Model 1: Model adjusted for age and sex

Model 2: Model 1 + ethnicity + BMI + T2D + Smoking status (never, past, current) + Alcohol consumption (<1, 1-7,>7 drinks/week) + Education (low, medium, high) + SBP

Model 3: Model 2 + ferritin + hemoglobin + MCV

Model 4: Model 3 + TC / HDL-C ratio + glucose

Model 5: Model 4 + cancer history + CVD history

Model 6: Model 5 + eGFR + UAE

Model 7: Model 6 + ALT + AST + ALP + GGT

© 2020 Flores-Guerrero JL et al. *JAMA Network Open.*

eTable 6. Prospective Associations of Plasma Concentration of Vitamin B_{12} With Risk of All-Cause Mortality in All Individuals With Available Information

	**Vitamin B_{12} Per 1 SD Increment**		**Q1**	**Q2+Q3**		**Q4**	
Participants, *n*	5629		1407	2813		1409	
Events, *n*	233		44	113		76	
	**HR (95 % CI)**	***P*-Value**		**HR (95 % CI)**	***P*-Value**	**HR (95 % CI)**	***P*-Value**
Crude Model	1.23 [1.08;1.40]	0.001	(ref)	1.27 [0.90;1.80]	0.17	1.71 [1.18;2.48]	0.004
Model 1	1.20 [1.06;1.36]	0.004	(ref)	1.26 [0.89;1.79]	0.18	1.67 [1.15;2.42]	0.006
Model 2	1.25 [1.09;1.42]	0.001	(ref)	1.28 [0.89;1.83]	0.18	1.81 [1.22;2.68]	0.002
Model 3	1.24 [1.08;1.43]	0.002	(ref)	1.33 [0.91;1.95]	0.14	1.75 [1.15;2.68]	0.009
Model 4	1.24 [1.07;1.43]	0.003	(ref)	1.32 [0.90;1.95]	0.15	1.71 [1.11;2.63]	0.01
Model 5	1.20 [1.04;1.39]	0.01	(ref)	1.32 [0.89;1.94]	0.16	1.64 [1.07;2.53]	0.02
Model 6	1.21 [1.04;1.41]	0.01	(ref)	1.35 [0.90;2.03]	0.14	1.79 [1.13;2.83]	0.01
Model 7	1.23 [1.05;1.44]	0.009	(ref)	1.32 [0.88;1.99]	0.18	1.80 [1.14;2.86]	0.01

Data are presented as hazard ratios (HRs) with 95% confidence intervals (CIs). For HR of Vitamin B_{12} per 1 SD increment, data was $\log_e$ transformed.

Model 1: Model adjusted for age and sex.

Model 2: Model 1 + ethnicity + BMI + T2D + Smoking status (never, past, current) + Alcohol consumption (<1, 1-7,>7 drinks/week) + Education (low, medium, high) + SBP

Model 3: Model 2 + ferritin + hemoglobin + MCV.

Model 4: Model 3 + TC/HDL-C ratio + glucose.

Model 5: Model 4 + cancer history + CVD history.

Model 6: Model 5 + eGFR + UAE.

Model 7: Model 6 + ALT + AST + ALP + GGT + B_{12} supplementation

© 2020 Flores-Guerrero JL et al. *JAMA Network Open.*

eTable 7. Prospective Associations of Plasma Concentration of Vitamin B_{12} With Risk of All-Cause Mortality After Excluding Individuals With Mild to Moderated Loss of Kidney Function

	**Vitamin B_{12} Per 1 SD Increment**		**Q1**	**Q2+Q3**		**Q4**	
Participants, *n*	5342		1333	2665		1344	
Events, *n*	180		34	89		57	
	**HR (95 % CI)**	***P*-Value**		**HR (95 % CI)**	***P*-Value**	**HR (95 % CI)**	***P*-Value**
Crude Model	1.20 [1.04;1.39]	0.01	(ref)	1.30 [0.87;1.92]	0.20	1.64 [1.07;2.51]	0.02
Model 1	1.19 [1.03;1.38]	0.01	(ref)	1.30 [0.88;1.93]	0.19	1.63 [1.07;2.50]	0.02
Model 2	1.19 [1.01;1.39]	0.03	(ref)	1.23 [0.82;1.85]	0.32	1.63 [1.04;2.55]	0.03
Model 3	1.20 [1.02;1.43]	0.03	(ref)	1.24 [0.80;1.91]	0.33	1.62 [1.00;2.62]	0.04
Model 4	1.20 [1.01;1.43]	0.03	(ref)	1.22 [0.78;1.89]	0.38	1.58 [0.97;2.58]	0.06
Model 5	1.17 [0.99;1.40]	0.06	(ref)	1.21 [0.78;1.88]	0.39	1.52 [0.93;2.50]	0.09
Model 6	1.19 [0.99;1.43]	0.06	(ref)	1.26 [0.79;2.01]	0.33	1.70 [1.01;2.87]	0.04
Model 7	1.18 [0.98;1.42]	0.07	(ref)	1.28 [0.80;2.05]	0.29	1.70 [1.00;2.89]	0.04

Data are presented as hazard ratios (HRs) with 95% confidence intervals (CIs). For HR of Vitamin B_{12} per 1 SD increment, data was $\log_e$ transformed.

Model 1: Model adjusted for age and sex.

Model 2: Model 1 + ethnicity + BMI + T2D + Smoking status (never, past, current) + Alcohol consumption (<1, 1-7,>7 drinks/week) + Education (low, medium, high) + SBP + homocysteine.

Model 3: Model 2 + ferritin + hemoglobin + MCV.

Model 4: Model 3 + TC/HDL-C ratio + glucose.

Model 5: Model 4 + cancer history + CVD history.

Model 6: Model 5 + eGFR + UAE.

Model 7: Model 6 + ALT + AST + ALP + GGT.

© 2020 Flores-Guerrero JL et al. *JAMA Network Open.*

eTable 8. Prospective Associations of Plasma Concentration of Vitamin B_{12} With Risk of All-Cause Mortality in a Design-Based Analysis

	**Vitamin B_{12} Per 1 SD Increment**		**Q1**	**Q2+Q3**		**Q4**	
Participants, *n*	5571		1390	2787		1394	
Events, *n*	226		41	112		73	
	**HR (95 % CI)**	***P*-Value**		**HR (95 % CI)**	***P*-Value**	**HR (95 % CI)**	***P*-Value**
Crude Model	1.36 [1.17;1.57]	<0.001	(ref)	1.19 [0.80;1.77]	0.40	2.06 [1.37;3.10]	<0.001
Model 1	1.32 [1.14;1.53]	<0.001	(ref)	1.19 [0.80;1.78]	0.38	1.98 [1.31;2.98]	0.001
Model 2	1.38 [1.18;1.62]	<0.001	(ref)	1.20 [0.79;1.82]	0.38	2.21 [1.43;3.43]	<0.001
Model 3	1.38 [1.17;1.63]	<0.001	(ref)	1.23 [0.80;1.89]	0.35	2.13 [1.34;3.38]	0.001
Model 4	1.33 [1.12;1.57]	0.001	(ref)	1.16 [0.75;1.80]	0.49	1.90 [1.19;3.05]	0.007
Model 5	1.30 [1.10;1.54]	0.002	(ref)	1.15 [0.74;1.79]	0.52	1.84 [1.15;2.97]	0.01
Model 6	1.33 [1.12;1.58]	0.001	(ref)	1.17 [0.75;1.84]	0.48	2.00 [1.22;3.25]	0.005
Model 7	1.33 [1.12;1.59]	0.001	(ref)	1.16 [0.74;1.81]	0.52	2.01 [1.23;3.30]	0.005

Data are presented as hazard ratios (HRs) with 95% confidence intervals (CIs). For HR of Vitamin B_{12} per 1 SD increment, data was $\log_e$ transformed.

Model 1: Model adjusted for age and sex.

Model 2: Model 1 + ethnicity + BMI + T2D + Smoking status (never, past, current) + Alcohol consumption (<1, 1-7,>7 drinks/week) + Education (low, medium, high) + SBP + homocysteine

Model 3: Model 2 + ferritin + hemoglobin + MCV.

Model 4: Model 3 + TC/HDL-C ratio + glucose.

Model 5: Model 4 + cancer history + CVD history.

Model 6: Model 5 + eGFR + UAE.

Model 7: Model 6 + ALT + AST + ALP + GGT + B_{12} supplementation

© 2020 Flores-Guerrero JL et al. *JAMA Network Open.*

eTable 9. All-Cause Mortality Relative Risks for Each Stratum of Smoking Behavior

	**All-cause mortality**			
	**Dead**	**Alive**	**Total**	**Proportion of deaths (all-cause mortality) (%)**
All participants [a]				
Q1 of Vitamin B_{12}	41	1349	1390	2.9
Q2+Q3 of Vitamin B_{12}	112	2675	2787	4.0
Q4 of Vitamin B_{12}	73	1321	1394	5.2
Participants who never smoked [b]				
Q1 of Vitamin B_{12}	7	372	379	1.8
Q2+Q3 of Vitamin B_{12}	13	765	778	1.7
Q4 of Vitamin B_{12}	12	408	420	2.9
Former smokers [c]				
Q1 of Vitamin B_{12}	25	556	581	4.3
Q2+Q3 of Vitamin B_{12}	61	1119	1180	5.2
Q4 of Vitamin B_{12}	42	575	617	6.8
Current smokers [d]				
Q1 of Vitamin B_{12}	9	400	409	2.2
Q2+Q3 of Vitamin B_{12}	37	759	796	4.6
Q4 of Vitamin B_{12}	19	323	342	5.6

a. Crude Relative Risk=5.2%/2.9%= 1.8.

b. Stratum-specific Relative Risk =2.9%/1.8%= 1.6.

c. Stratum-specific Relative Risk =6.8%/4.3%= 1.6.

d. Stratum-specific Relative Risk =5.6%/2.2%= 2.5.

© 2020 Flores-Guerrero JL et al. *JAMA Network Open.*

eTable 10. All-Cause Mortality Relative Risks for Each Stratum of Alcohol Consumption Behavior

	**All-cause mortality**			
	**Dead**	**Alive**	**Total**	**Proportion of deaths (all-cause mortality) (%)**
All Participants [a]				
Q1 of Vitamin B_{12}	41	1349	1390	2.9
Q2+Q3 of Vitamin B_{12}	112	2675	2787	4.0
Q4 of Vitamin B_{12}	73	1321	1394	5.2
Participants with alcohol intake<1 drinks/week [b]				
Q1 of Vitamin B_{12}	15	337	352	4.5
Q2+Q3 of Vitamin B_{12}	33	674	707	4.7
Q4 of Vitamin B_{12}	20	345	365	5.5
Participants with alcohol intake= 1-7 drinks/week [c]				
Q1 of Vitamin B_{12}	20	650	670	3.0
Q2+Q3 of Vitamin B_{12}	49	1316	1365	3.6
Q4 of Vitamin B_{12}	28	590	618	4.5
Participants with alcohol intake >7 drinks/week [d]				
Q1 of Vitamin B_{12}	15	337	352	4.3
Q2+Q3 of Vitamin B_{12}	33	674	707	4.7
Q4 of Vitamin B_{12}	20	345	365	5.5

a. Crude Relative Risk=5.2%/2.9%= 1.8.

b. Stratum-specific Relative Risk =5.5%/4.5%= 1.2.

c. Stratum-specific Relative Risk =4.5%/3.0%= 1.5.

d. Stratum-specific Relative Risk =5.5%/4.3%= 1.3.

© 2020 Flores-Guerrero JL et al. *JAMA Network Open.*

eTable 11. All-Cause Mortality Relative Risks for Each Stratum of Age

	**All-cause mortality**			
	**Dead**	**Alive**	**Total**	**Proportion of deaths (all-cause mortality) (%)**
All Participants [a]				
Q1 of Vitamin B_{12}	41	1349	1390	2.9
Q2+Q3 of Vitamin B_{12}	112	2675	2787	4.0
Q4 of Vitamin B_{12}	73	1321	1394	5.2
Participants <65 years old [b]				
Q1 of Vitamin B_{12}	12	1089	1101	1.1
Q2+Q3 of Vitamin B_{12}	31	2184	2215	1.4
Q4 of Vitamin B_{12}	20	1061	1081	1.9
Participants with ≥65 years old [c]				
Q1 of Vitamin B_{12}	29	260	289	10.0
Q2+Q3 of Vitamin B_{12}	81	491	572	14.2
Q4 of Vitamin B_{12}	53	260	313	16.9

a. Crude Relative Risk=5.2%/2.9%= 1.8.

b. Stratum-specific Relative Risk =1.9%/1.1%= 1.7.

c. Stratum-specific Relative Risk =16.9%/10%= 1.7.

.

© 2020 Flores-Guerrero JL et al. *JAMA Network Open.*

eTable 12. Prospective Associations of Plasma Concentration of Vitamin B_{12} With Risk of Cancer Mortality

	**Vitamin B_{12} Per 1 SD Increment**		**Q1**	**Q2+Q3**		**Q4**	
Participants, *n*	5571		1390	2787		1394	
Events, *n*	172		41	76		55	
	**HR (95 % CI)**	***P* Value**		**HR (95 % CI)**	***P* Value**	**HR (95 % CI)**	***P* Value**
Crude Model	1.14 [0.98;1.33]	0.08	(ref)	0.94 [0.64;1.37]	0.73	1.36 [0.91;2.04]	0.13
Model 1	1.13 [0.97;1.31]	0.10	(ref)	0.92 [0.63;1.35]	0.67	1.33 [0.88;1.99]	0.17
Model 2	1.12 [0.96;1.32]	0.15	(ref)	0.86 [0.58;1.28]	0.45	1.32 [0.86;2.02]	0.20
Model 3	1.12 [0.94;1.33]	0.20	(ref)	0.88 [0.58;1.33]	0.53	1.26 [0.80;2.00]	0.31
Model 4	1.10 [0.92;1.31]	0.29	(ref)	0.86 [0.56;1.30]	0.47	1.18 [0.74;1.87]	0.49
Model 5	1.09 [0.91;1.30]	0.35	(ref)	0.83 [0.55;1.27]	0.39	1.17 [0.73;1.86]	0.51
Model 6	1.11 [0.92;1.33]	0.26	(ref)	0.87 [0.57;1.35]	0.54	1.22 [0.75;1.98]	0.43
Model 7	1.13 [0.93;1.36]	0.21	(ref)	0.88 [0.57;1.36]	0.55	1.28 [0.78;2.09]	0.32

Data are presented as hazard ratios (HRs) with 95% confidence intervals (CIs). For HR of Vitamin B_{12} per 1 SD increment, data was $\log_e$ transformed.

Model 1: Model adjusted for age and sex.

Model 2: Model 1 + ethnicity + BMI + T2D + Smoking status (never, past, current) + Alcohol consumption (<1, 1-7,>7 drinks/week) + Education (low, medium, high) + SBP + homocysteine.

Model 3: Model 2 + ferritin + hemoglobin + MCV.

Model 4: Model 3 + TC/HDL-C ratio + glucose.

Model 5: Model 4 + cancer history + CVD history.

Model 6: Model 5 + eGFR + UAE.

Model 7: Model 6 + ALT + AST + ALP + GGT.

© 2020 Flores-Guerrero JL et al. *JAMA Network Open.*

eTable 13. Prospective Associations of Plasma Concentration of Vitamin B_{12} With Risk of Cardiovascular Mortality

	**Vitamin B_{12} Per 1 SD Increment**		**Q1**	**Q2+Q3**		**Q4**	
Participants, *n*	5571		1390	2787		1394	
Events, *n*	53		8	29		16	
	**HR (95 % CI)**	***P* Value**		**HR (95 % CI)**	***P* Value**	**HR (95 % CI)**	***P* Value**
Crude Model	1.20 [0.91;1.57]	0.19	(ref)	1.79 [0.82;3.91]	0.14	1.97 [0.84;4.61]	0.11
Model 1	1.20 [0.92;1.58]	0.18	(ref)	1.81 [0.83;3.97]	0.13	1.99 [0.85;4.66]	0.11
Model 2	1.24 [0.93;1.66]	0.14	(ref)	2.20 [0.97;4.98]	0.05	2.07 [0.80;5.32]	0.13
Model 3	1.21 [0.90;1.64]	0.21	(ref)	2.15 [0.94;4.92]	0.07	1.91 [0.72;5.04]	0.19
Model 4	1.20 [0.88;1.62]	0.25	(ref)	2.26 [0.97;5.27]	0.05	1.79 [0.66;4.86]	0.25
Model 5	1.16 [0.85;1.59]	0.34	(ref)	2.31 [0.98;5.42]	0.05	1.62 [0.58;4.54]	0.35
Model 6	1.07 [0.76;1.51]	0.69	(ref)	1.88 [0.79;4.51]	0.15	1.37 [0.46;4.03]	0.56
Model 7	1.06 [0.75;1.51]	0.74	(ref)	1.79 [0.74;4.32]	0.19	1.33 [0.45;3.95]	0.60

Data are presented as hazard ratios (HRs) with 95% confidence intervals (CIs). For HR of Vitamin B_{12} per 1 SD increment, data was $\log_e$ transformed.

Model 1: Model adjusted for age and sex.

Model 2: Model 1 + ethnicity + BMI + T2D + Smoking status (never, past, current) + Alcohol consumption (<1, 1-7,>7 drinks/week) + Education (low, medium, high) + SBP + homocysteine.

Model 3: Model 2 + ferritin + hemoglobin + MCV.

Model 4: Model 3 + TC/HDL-C ratio + glucose.

Model 5: Model 4 + cancer history + CVD history.

Model 6: Model 5 + eGFR + UAE.

Model 7: Model 6 + ALT + AST + ALP + GGT.

© 2020 Flores-Guerrero JL et al. *JAMA Network Open.*
